# Eco-Friendly Protection of Copper via Self-Assembled Monolayer Films: The Critical Effect of Pollen Source Region on Anti-Corrosion Behavior

**DOI:** 10.1021/acsomega.6c03873

**Published:** 2026-06-19

**Authors:** Ramazan Solmaz, Ece Altunbaş Şahin, Sinan Bayındır, Yeşim Aydın Dursun, İbrahim Halil Gecibesler, Mustafa Doğrubaş, Nevzat Çağlayan, İnan Dursun, İbrahim Şahin, Handan Yüksel, İbrahim Y. Erdoğan, Gülfeza Kardaş

**Affiliations:** † Bingöl University, Health Sciences Faculty, Occupational Health and Safety Department, 12000 Bingöl, Türkiye; ‡ Bingöl University, Genç Vocational School, Property Protection and Security Department, Civil Defense and Firefighting Program, 12000 Bingöl, Türkiye; § Bingöl University, Science and Letters Faculty, Chemistry Department, 12000 Bingöl, Türkiye; ∥ Bingöl University, Graduate School of Natural and Applied Sciences, Chemistry Department, 12000 Bingöl, Türkiye; ⊥ Bingöl University, Graduate School of Natural and Applied Sciences, Occupational Health and Safety Department, 12000 Bingöl, Türkiye; # Bingöl University, Vocational School of Food, Agriculture, and Livestock, Plant and Animal Production Department, Beekeeping Program, 12000 Bingöl, Türkiye; ∇ Bingöl University, Beekeeping Research, Development, Application and Research Center, 12000 Bingöl, Türkiye; ○ Çukurova University, Arts and Sciences Faculty, Chemistry Department, 01330 Adana, Türkiye; ◆ Bingöl University, Central Laboratory Application and Research Center, 12000 Bingöl, Türkiye

## Abstract

*Background*: This study initially investigates the corrosion inhibition efficiency of SAM films (self-assembled monolayer) derived from Bingöl pollen collected from various source regions on copper in a 3.5% NaCl environment. The films were designated as B-pollen­(X)/SAM, where X represents the specific region. *Methods*: For a comprehensive understanding, the resulting SAM film characteristics on the copper substrate were analyzed using complementary techniques, including Scanning Electron Microscopy (SEM), Energy Dispersive X-ray Spectroscopy (EDX), and contact angle measurements. Subsequently, a range of electrochemical techniques was employed to precisely quantify how regional variations in pollen source affect copper corrosion protection. The SAM film derived from Karlıova region pollen exhibited the highest corrosion inhibition efficiency on copper in 3.5% NaCl. *Significant Findings*: Overall, this study establishes that geographical source is a critical factor for optimizing the efficiency of pollen-derived SAM films, providing a sustainable and cost-effective pathway for the development of highly effective metallic protection systems.

## Graphical abstract

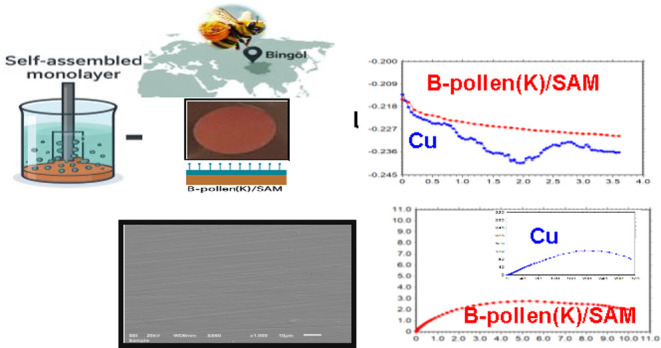

## 1 Introduction

The rapid advancement of industrial technology, from the Industrial Revolution to the modern era, has greatly increased the prevalence and severity of metal corrosion, transforming it from a localized technical problem into a global industrial issue of high importance.^1–3^ Metal corrosion has long been recognized as a significant barrier to industrial progress, presenting complex challenges that demand attention at both national and global levels.^4–6^ This pervasive phenomenon not only incurs substantial economic losses but also introduces critical safety risks, with implications extending beyond financial damage to the well-being of workers and communities.^7–10^ Corrosion degrades the mechanical properties of metallic materials—such as strength, flexibility, and resilience—thereby compromising the structural integrity of components. Additionally, it increases friction between interacting parts, reduces electrical and optical performance, and undermines the overall functionality and reliability of metals in various applications.^11–16^ If left unchecked, corrosion can cause irreversible damage, leading to long-term challenges that become increasingly difficult and costly to remedy. Consequently, advancing the understanding of corrosion mechanisms and developing effective mitigation strategies remain central priorities in materials science and engineering research and innovation. Copper is a versatile metal widely used across many sectors, including electronics, construction, transportation, and the chemical industry.^17–21^ Its distinct combination of properties, such as excellent electrical and thermal conductivity, high ductility, and affordability, makes it an essential material for modern engineering applications. In the microelectronics industry, copper is especially valued for its use in flexible circuits, connectors, and as an efficient cooling medium for heat-generating components such as processors.^22–27^ Because of these widespread uses, global production has steadily increased, reaching 48,000 t in 2022.^28^

Although copper exhibits strong corrosion resistance, it remains vulnerable to degradation in environments containing Cl ions. The corrosion of copper not only compromises structural integrity and equipment safety but also causes substantial economic losses and poses hazards to human life.^29,30^ This persistent issue motivates ongoing advances in the development of innovative and effective protection methods to mitigate its impact. Although complete prevention of corrosion in copper and its alloys is unattainable, researchers increasingly focus on strategies to reduce the corrosion rate. This ongoing research aims to slow the corrosion process, thereby extending the service life and performance of copper-based materials in diverse industrial applications. Efforts focus on elucidating corrosion mechanisms and developing techniques to enhance the resistance of copper and its alloys, helping them maintain functional integrity over time.^31–35^

Corrosion-control strategies, including cathodic and anodic protection,^36,37^ metallic coatings and self-assembled monolayer (SAM) films, and corrosion inhibitors,^38–45^ are widely employed to protect metals from corrosion, thereby enhancing their durability and prolonging service life.

While organic inhibitors containing heteroatoms such as S, O, and N, typically as electron-pair-donating compounds (e.g., triazoles, imidazoles, thiophenes), are widely used for corrosion protection, environmental concerns regarding these inhibitors have prompted researchers to explore more sustainable alternatives.^46–50^

The SAM method involves fundamental structural units spontaneously organizing into a well-ordered structure. SAMs are densely packed, ordered, and stable molecular assemblies^51^ that form through spontaneous adsorption of inhibitor molecules onto the metal substrate via chemical bonds. Therefore, SAMs can protect the substrate from corrosion in corrosive environments.^52–55^ Consequently, they offer a cost-effective and efficient method for preparing high-density films to inhibit copper corrosion. Compared to conventional corrosion inhibition approaches, SAMs have been widely researched due to their lower dosage, greater surface coverage, fewer defects, and anticorrosive performance.^56–60^

Building on the two synergistic effects, the preparation of SAM films from eco-friendly natural products (e.g., bee products) and their application in corrosion studies remains relatively unexplored in the literature. A survey by Solmaz et al.^61^ examined self-assembled monolayer (SAM) films prepared from Bingöl propolis (B-PP/SAM) for copper protection in 3.5% NaCl. B-PP samples from Bingöl’s northern regions (Adaklı, Kiğı, Yayladere, and Yedisu) were used to form SAM films under varied conditions, including different solvents, concentrations, and film-formation times. Highly adherent and homogeneous B-PP/SAM films were successfully prepared, with the most effective protection achieved using ethanol as the solvent, 1000 ppm B-PP, and a 24 h formation time. Electrochemical techniques such as electrochemical impedance spectroscopy (EIS), linear polarization technique (LPR), and potentiodynamic polarization (PDP) measurements showed inhibition efficiencies exceeding 95%. This study demonstrates the potential of natural products in forming efficient SAM films for corrosion protection.

The protective effects of SAM films formed from pollen, another bee-derived product,^62^ on copper were recently investigated in detail by our research team. The optimal conditions for pollen were identified as a concentration of 1000 ppm, water as the solvent, and a film-formation time of 24 h. The pollen used in the study was sourced from the northern regions of Bingöl Province, and extracts were subsequently prepared. This is the only reported study on pollen-based SAM films, and further studies have not been reported. The previous study served purely as a “proof-of-concept” to establish the optimal physical film-forming parameters (solvent type, concentration, and assembly time) using a single pollen batch. The research focused on optimizing parameters such as solvent, time, and concentration using a representative pollen sample from the Northern region of Bingöl to show that pollen can indeed form a SAM. In contrast, the current study introduces the higher-level concept of “Geographical Tracing of Biomaterials”. It should be emphasized that variations in topography and climate naturally dictate the local flora, which fundamentally alters the phytochemical profile (e.g., phenolics, flavonoids, and proteins) of the pollen. This demonstrates that geographical origin is a critical design parameter, not just a random variable and also affects the quality as well as the electrochemical properties of the surface film. This article is the first to suggest that the geographical origin is an essential parameter for the optimization of biobased films. By comparing the films from five different areas, we go beyond the traditional material science approach to propose the design of sustainable materials. The chemical compositions of pollen samples from all regions were analyzed comprehensively using Exactive Plus Orbitrap HPLC-HRMS, and the detailed results, alongside the corresponding standards, will be reported in subsequent studies.

Building upon our previous work, in which the SAM films of pollen collected from the northern region of Bingöl were optimized for copper protection in 3.5% NaCl solution,^62^ the present study expands this research by systematically investigating the influence of geographical origin on SAM film performance. In a previous study,^62^ our research group reported the formation and characterization of pollen-derived SAMs using pollen collected from a single geographical region. That work primarily focused on demonstrating the feasibility of pollen-based SAM formation and its basic corrosion-protective behavior. The present study, although originating from the same research project, addresses a distinct and complementary research question by systematically comparing pollens collected from different geographical regions and investigating how geographical origin influences SAM formation and corrosion protection performance. Therefore, the current manuscript extends the previous findings by introducing a comparative, origin-dependent perspective, providing additional insight into the structure–property relationship of pollen-based SAM coatings and enhancing the overall understanding of their applicability in corrosion protection. In this new work, using the formerly reported optimized parameters, SAM films were prepared from pollen samples harvested from five distinct regions of Bingöl Province, Northern Region (Adaklı, Kiğı, Yayladere, and Yedisu), Solhan, Genç, Central, and Karlıova, each characterized by unique topographic and climatic conditions. Variations in geographic and climatic factors will naturally cause variations in local flora and the chemical makeup of the collected pollen, which included differences in content and ratios of phenolic, flavonoid, and protein fractions, which are known contributors to adsorption and the SAM on metal surfaces. As such, this study is the first to determine the relationship between the origin of pollen and the quality, morphology, and corrosion inhibition of resulting SAM films. The results should provide new ideas about the impact of regional biodiversity and the environment on the protective stability of biopolymer-derived SAM films on copper and provide a new way to think conceptually in the design of sustainable and nature-inspired corrosion inhibition approaches. A detailed analysis of the composition and physicochemical properties of the samples will be addressed in a subsequent study.

The primary objective of this study is to examine how SAM films on copper, derived from pollen samples harvested from various regions of Bingöl Province (Figure 1), affect the corrosion rate in 3.5% NaCl. A range of electrochemical methods, including *E*_ocp_–*t*, EIS, LPR, and PDP methods, were employed to evaluate the protective performance of SAM films on the copper surface in corrosive media. The findings provide a foundational framework to advance the development and design of novel self-assembling molecules derived from natural biobased materials.

**Figure 1:**
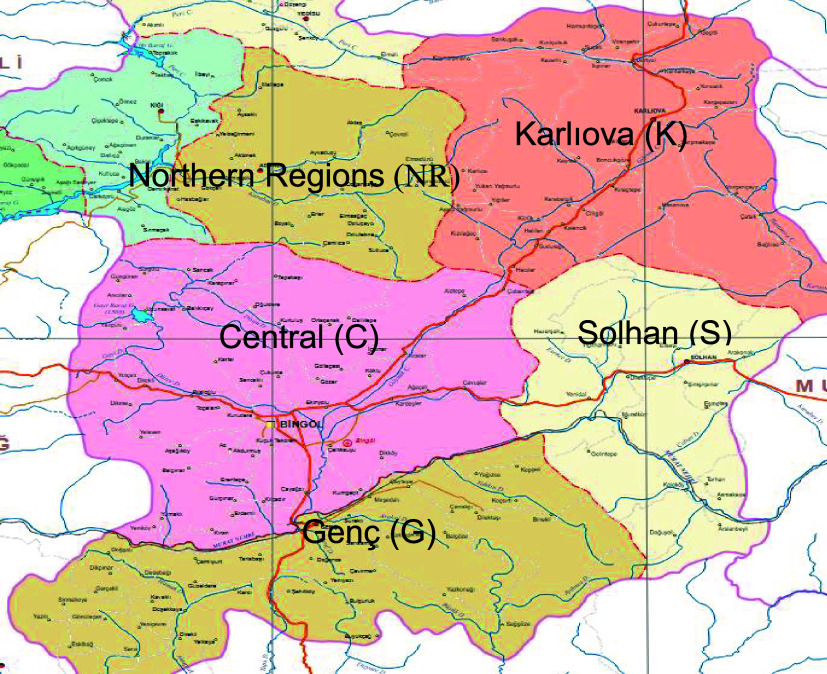
Regions of Bingöl Province where pollen is harvested (Northern regions) (NR), Genç (G), Central (C), Solhan (S) and Karlıova (K), (The map presented in this figure was originally reference to^93^ and revised by the authors, and no external copyright permission is required).^93^

## 2 Materials and Methods

### 2.1 Preparation of Working Electrodes

The copper electrodes used as working electrodes for electrochemical tests were cut from a cylindrical rod, with a surface area of 0.0707 cm^2^ (3 mm diameter). The copper rods, approximately 5 cm in length, were embedded in a polyester block, providing one exposed working face, while a copper wire was affixed on the opposite side to ensure electrical contact. Copper samples used for characterization had a 3 mm diameter and were about 5 mm in length. Copper electrodes prepared by this method were also used to prepare SAM-coated working electrodes.

Pollen samples harvested from various regions of Bingöl province, Northern Region (NR) (Adaklı, Kiğı, Yayladere, and Yedisu), Solhan (S), Genç (G), Central (C) and Karlıova (K), were used to prepare electrodes modified with SAM films. The preparation of B-pollen/SAM electrodes in this study was based on our previous work.^62^ The best results were achieved with a 24 h film formation time and 1000 ppm pollen dissolved in water.

SAM films prepared in this study were formed in an aqueous solution containing 1000 ppm pollen extract. Prior to the assembly process, nitrogen gas was purged through the cell for 30 min to remove dissolved oxygen. Copper substrates were mechanically polished using successive grades of sandpaper (100–2000 grit) and subsequently etched in 7 M HNO_3_ for approximately 10 s. The electrodes were then thoroughly rinsed with distilled water and ethanol, followed by ultrasonic cleaning in ethanol for about 10 s. After cleaning, the copper electrodes were placed into the prepared cell, and nitrogen gas was purged continuously.

### 2.2 Electrochemical Measurements

The corrosion tests of uncoated and SAM-coated copper samples were conducted in a 3.5% NaCl solution. All electrochemical experiments were carried out using a CHI6092E electrochemical workstation. Experiments were performed at 298 K using a thermostat bath (Nüve NB 20) set to 298 K. A conventional three-electrode electrochemical cell was employed. A 10 mm × 10 mm × 1 mm platinum sheet served as the counter electrode. A commercially available Ag/AgCl reference electrode (3 M KCl) was utilized in the experiments; all potentials were recorded relative to the reference electrode.

A 3.5% NaCl solution, one of the most commonly used corrosive electrolytes to simulate seawater conditions, was selected in order to accelerate laboratory-scale corrosion experiments. Examination of the Pourbaix diagram indicates that under these conditions, copper exists in an active dissolution region and is therefore susceptible to corrosion. At the outset, the electrodes were immersed in a 3.5% NaCl solution for 1 h, and the change in *E*_ocp_ over the duration of immersion was recorded. EIS measurement was performed at steady state *E*_ocp_ over a frequency range from 100 kHz to 10 mHz, applying an AC amplitude of 10 mV. These measurements were also performed after various exposure times up to 120 h.

The EIS data were fitted and analyzed using Zview software and relevant parameters were derived. Each experiment was performed at least three replicates to verify the consistency of the data. The η_R_% value based on polarization resistance was calculated using the following equation

1 $$\eta_{R}=\left(\frac{R_{p}^{\prime }-R_{p}}{R_{p}^{\prime }}\right)\times 100$$

Here, *R*_p_ represents the polarization resistance of the bare copper electrode, while *R*′_p_ refers to the polarization resistance of the SAM-functionalized copper electrode.

Anodic PDP measurements of bare copper and B-pollen­(K)/SAM electrode were recorded in 3.5% NaCl solution following 120 h of exposure. The curves were recorded by scanning the electrode potential from *E*_ocp_ to +1.0 mV at a sweep rate of 1.0 mV s^–1^. The related electrochemical parameters, such as the corrosion potential (*E*_corr_), the corrosion current density (*i*_corr_), anodic Tafel slopes (*b*_a_), the current densities at +0.100 V (*i*_0.100V_) and +0.150 V (*i*_0.150V_) overpotentials, and the corrosion inhibition efficiency (η_i_%) values were derived from these curves. The data were compared to those recorded for shorter exposure times (1 h).

The following formula was employed to calculate the η_i_% values based on the current densities

2 $$\eta_{i}\%=\left(\frac{i_{\mathrm{cor}}^{\prime }-i_{\mathrm{cor}}}{i_{\mathrm{cor}}^{\prime }}\right)\times 100$$

Here, *i*′_cor_ and *i*_cor_ represent the corrosion current densities derived for uncoated and SAM-modified Cu electrodes, respectively.

LPR measurements were conducted to assess the behavior of SAM-coated electrodes, assembled under different conditions outlined earlier, following 1 h immersion of the working electrode in 3.5% NaCl solution to establish a steady-state condition. Equation 1 was used to calculate the η_R_% values. These measurements were also performed after various exposure times up to 120 h.

### 2.3 Surface Characterization Tests

The surface morphologies of both unmodified and SAM-modified copper electrodes were analyzed using SEM and AFM. The SEMs of all electrodes were captured using a Jeol model (JEOL 6510) device. Elemental analysis was carried out using an integrated EDX-SEM system. The AFM analysis was carried out using Nanotechnology Park Systems XE-100 AFM devices.

The contact angle measurements for unmodified copper and SAM-modified copper electrodes were performed based on the sessile water drop method using a contact angle measuring system; the volume of the water droplet is about 2 μL. The experiments were conducted at room temperature. To calculate the average contact angle, at least three measurements were taken at different points over each specimen, and the corresponding mean values were computed.

## 3 Results and Discussion

### 3.1 Optimization of Pollen Origin

The EIS technique is one of the most commonly used to evaluate the performance and compatibility of SAM films on metal surfaces, particularly in corrosive behavior. This technique is preferred because it allows direct comparison of the performance between modified and un-modified electrodes.^63–65^ EIS parameters were obtained through fitting and analysis of the experimental data using ZView software. For this text analysis, the electrical equivalent circuit diagrams (EECD) provided in Figure 2b,2c were primarily used. Herein, *R*_ct_ is the charge transfer resistance, *R*_f_ is the film resistance formed on the metals surface, CPE_f_ and CPE_dl_, which contain *Y*_0_ and *n*, refer to constant phase elements corresponding to film and double layer, respectively.^66,67^ “*n*” is a measure of surface inhomogeneity, indicating deviation from the behavior of an ideal capacitor. The total resistance (*R*_p_) was considered as polarization resistance.

**Figure 2:**
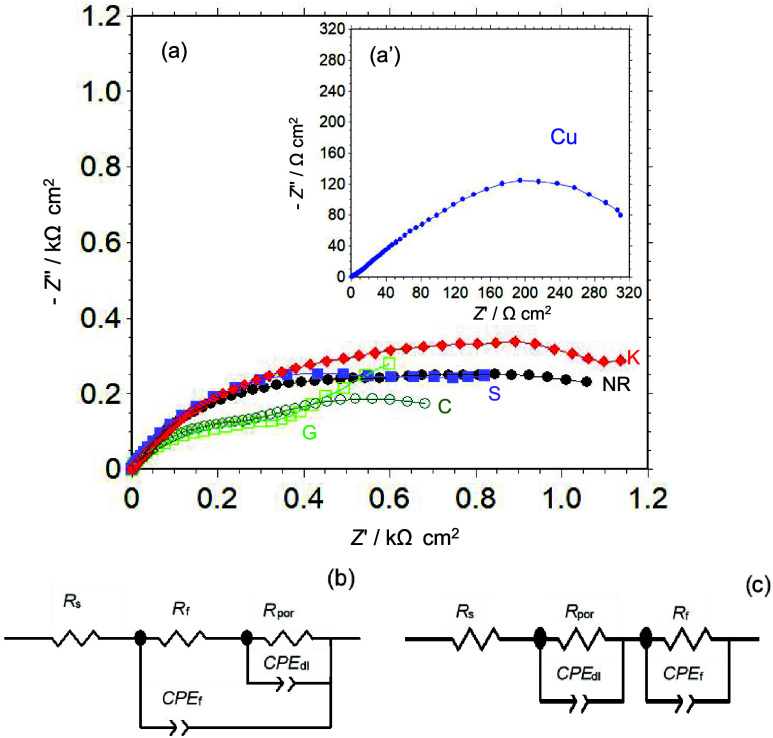
EIS curves of the uncoated (a′) and the B-pollen/SAM-modified copper electrodes (a), which were prepared using pollen harvested from different regions of Bingöl, after exposure to %3.5 NaCl solution for 1 h (B-pollen­(NR)/SAM) (●), B-pollen­(G)/SAM (◊), B-pollen­(C)/SAM (○), B-pollen­(S)/SAM (■) and B-pollen­(K)/SAM (⧫), electrical equivalent circuit diagrams proposed for bare copper/NaCl solution (b) and B-pollen/SAM/solution interface (c).

The Nyquist plots in Figure 2a,2a′ correspond to SAM-coated copper electrodes, which were prepared using pollen harvested from different regions of Bingöl, and uncoated copper electrodes. The data were obtained in 3.5% NaCl medium at 298 K for 1 immersion. The Nyquist plot of the uncoated copper electrode (The inset in Figure 2, 2a′) indicates the presence of two distinct time constants. The first time constant, appearing in the high and medium frequency regions, is related to CPE_dl_–*R*_ct_, while the second time constant, observed in the low frequency region, corresponds to *CPE*_f_–*R*_f_. The *R*_p_ value of the uncoated Cu electrode was 320 Ω cm^2^.

Figure 2a shows the Nyquist plots of the B-pollen­(SAM)-modified electrodes, which display similar features. However, the magnitudes of the time constants differ and are significantly greater than those of the bare copper. The capacitive loops are depressed with centers below the real axis, even though they appear to be semicircular. Deviations of this nature, commonly referred to as frequency dispersion, are attributed to irregularities or heterogeneities of the solid surface.^68^ Table 1 presents relevant EIS parameters, including CPE_dl_, *R*_p_, *n* value, and *n*_R_% values. As presented in Table 1, the CPE_dl_ and *Y*_0_ values are higher for the uncoated electrode than those of the SAM-modified electrodes. This suggests that the SAM film interacts with the electrode surface, thereby reducing corrosion on exposed areas of the electrode.^62^ It is the different types of phytochemicals, such as flavonoids and phenolics, derived from the flora of different regions, such as Karlıova, which directly affect the compactness of the formed SAMs. The increase in *R*_f_ and *R*_ct_, as well as the decrease in CPE_dl_, is the fundamental evidence supporting the fact that complex phytochemicals derived from the pollens of the respective regions effectively replace the water molecules at the interface. Pollens collected from different altitudes and climatic conditions, such as those in Karlıova, have different types of phytochemicals. As a result, the formed SAMs differ in compactness, which is the reason for the significant variation in the *R*_ct_ and CPE_f_ values. Moreover, the high values of the *R*_f_ and the *R*_ct_, which were achieved with the pollen extract derived from the Karlıova region, are evidence for the fact that this region exhibits superior performance with the densest molecular film against the aggressive chloride ions. Data fitting was performed for all EIS measurements; however, to maintain visual clarity and avoid overcrowding the main text, only the representative fitting curves for bare copper and B-pollen­(K) samples are displayed. The complete set of fitting files and curves for all samples is provided in the Supporting Information (Figure S1). To validate the accuracy of the electrochemical modeling, the calculated χ2 values for all fitted data are summarized in Tables 1 and 3, confirming a high degree of correlation between the experimental and simulated results.

**Table 1:** Electrochemical Parameters Derived from EIS and LPR Measurements for the Uncoated and B-pollen/SAM-Modified Copper Electrodes, Prepared Using Pollen Harvested from Different Regions of Bingöl, after Immersion in 3.5% NaCl for 1 h

		CPE_dl_			CPE_f_						
Electrodes	*R*_ct_ (Ω cm^2^)	*Y* ^o^ (10^–6^/s*^n^* Ω^–1^ cm^–2^)	*n*_dl_	*R*_f_ (Ω cm^2^)	*Y* ^o^ (10^–6^/s*^n^* Ω^–1^ cm^–2^)	*n*_f_	*n*_R_%	χ2 × 10^–4^	*R*_p_	^a^ *R*_p_	^b^ *n*_R_%
Uncoated Copper	3.31	2425	0.730	350	12615	0.540	-		353.3	188	-
B-pollen(NR)/SAM	2841	169	0.821	9827	477	0.581	97.2 ± 0.008	28	12,668	8337	97.7 ± 0.032
B-pollen(G)/SAM	3344	1047	0.750	5708	1272	0.710	96.1 ± 0.031	650	9052	7692	97.8 ± 0.032
B-pollen(C)/SAM	1644	80	0.800	5998	421.9	0.740	95.4 ± 0.028	240	7642	7856	97.6 ± 0.024
B-pollen(S)/SAM	4796	1595	0.750	8197	1126	0.690	97.3 ± 0.042	51	12,993	8340	97.7 ± 0.039
B-pollen(K)/SAM	2260	2729	0.750	13,377	281	0.550	97.7 ± 0.105	5	15,637	14154	98.7 ± 0.058

a *R*_p_: Calculated from LPR.

b η%: calculated from LPR

To further illustrate and understand the nature of the interfacial film, it is also important to note that the results obtained in this study through EIS measurements provided indirect evidence for the presence of an adsorbed organic film rather than effects due to surface morphologies as discussed in the characterization studies. This is particularly true in terms of the significant decrease in CPE_dl_ values for all electrodes modified with SAMs, which indicates that there is indeed a decrease in the value of the effective dielectric constant at the metal/electrolyte interface. This is normally associated with the replacement of water molecules at the interface with organic compounds. At the same time, the appearance and increase in the value of the film resistance component (*R*_f_) in the equivalent circuit also indicated that there is indeed a film that acts as a barrier for charge and ionic transport. These changes in electrochemical parameters (*R*_p_, CPE_dl_, and *R*_f_) are indeed associated with the presence of a protective organic film derived from pollen constituents.

The *R*_p_ values differ significantly among SAM films prepared using pollen samples harvested from various regions. Importantly, the SAM structure in this study arises from molecular-scale adsorption of compounds derived from pollen, rather than from intact pollen particles. All the modified electrodes exhibit higher *R*_p_ values than that of the uncoated copper electrode. The *n*_R_% values were between 97.74%–98.67%. The highest efficiency (98.67%) was obtained for the B-pollen­(K)/SAM electrodes. This result for B-pollen­(K)/SAM is highly consistent with that obtained from the LPR data (Table 1).

Figure 3 shows SEM images, surface photographs of the films, and EDX-mapping of the B-pollen/SAM electrodes, which were prepared using pollen harvested from different regions of Bingöl. As seen from Figure 3, the SEM analyses clearly show the formation of a surface film on all samples, which differs significantly among regions. The highest-quality, compact and adherent film was observed for the pollen harvested in the Karlıova region. It is worth mentioning that in this work, although the size of the pollen particles is in the micron order, it should be noted that the SAM film is not formed from the whole pollen grains, but from the water-extracted molecular constituents of the pollen, such as flavonoids, amino acids, and phenolic compounds, which can adsorb on the Cu surface at the molecular level. Surface photographs clearly show assembled films with different colors and qualities. Compared to bare Cu, apparently, the films were engineered for all samples, and their quality is dependent on the origin of the pollen. The distribution of S element (EDX mapping image) of the same surfaces, namely the distribution of the pollen-SAM films, is presented in Figure 3. The EDX data support these results and show homogeneous films on the surface, which is most pronounced for Karlıova pollen.

**Figure 3:**
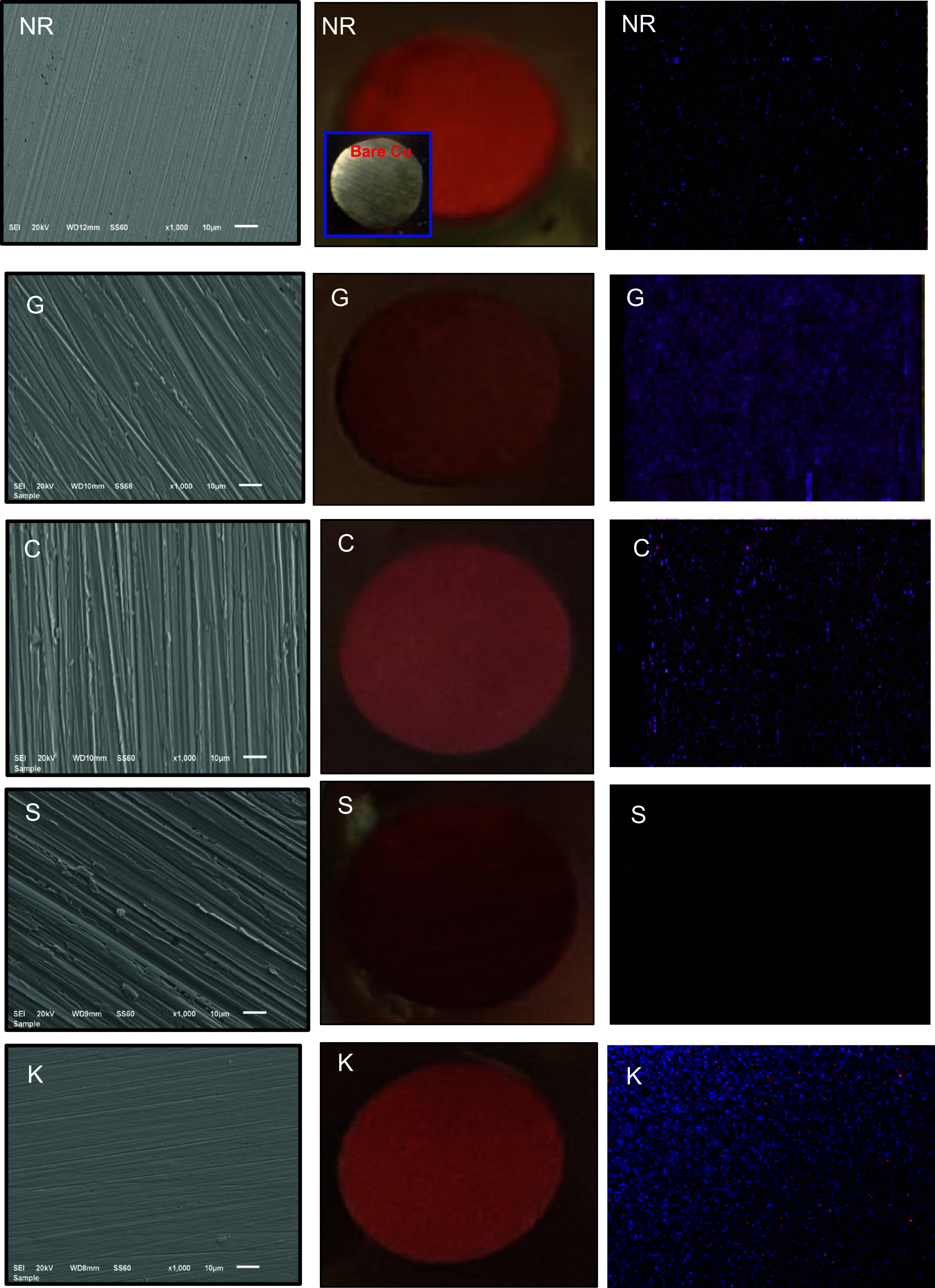
SEM images, surface photographs and EDX-mapping images (distribution of S element) of the B-pollen/SAM-modified copper electrodes, which were prepared using pollen harvested from different regions of Bingöl (B-pollen­(NR)/SAM (NR), B-pollen­(G)/SAM (G), B-pollen­(C)/SAM (C), B-pollen­(S)/SAM (S) and B-pollen­(K)/SAM (K)).

AFM images of bare copper and B-pollen/SAM electrodes, which were prepared using pollen harvested from different regions of Bingöl, are shown in Figure 4. The data are highly consistent with SEM-EDX results and confirm the formation of films on the surface for each pollen. Apparently, the best-quality film was assembled for the Karlıova pollen.

**Figure 4:**
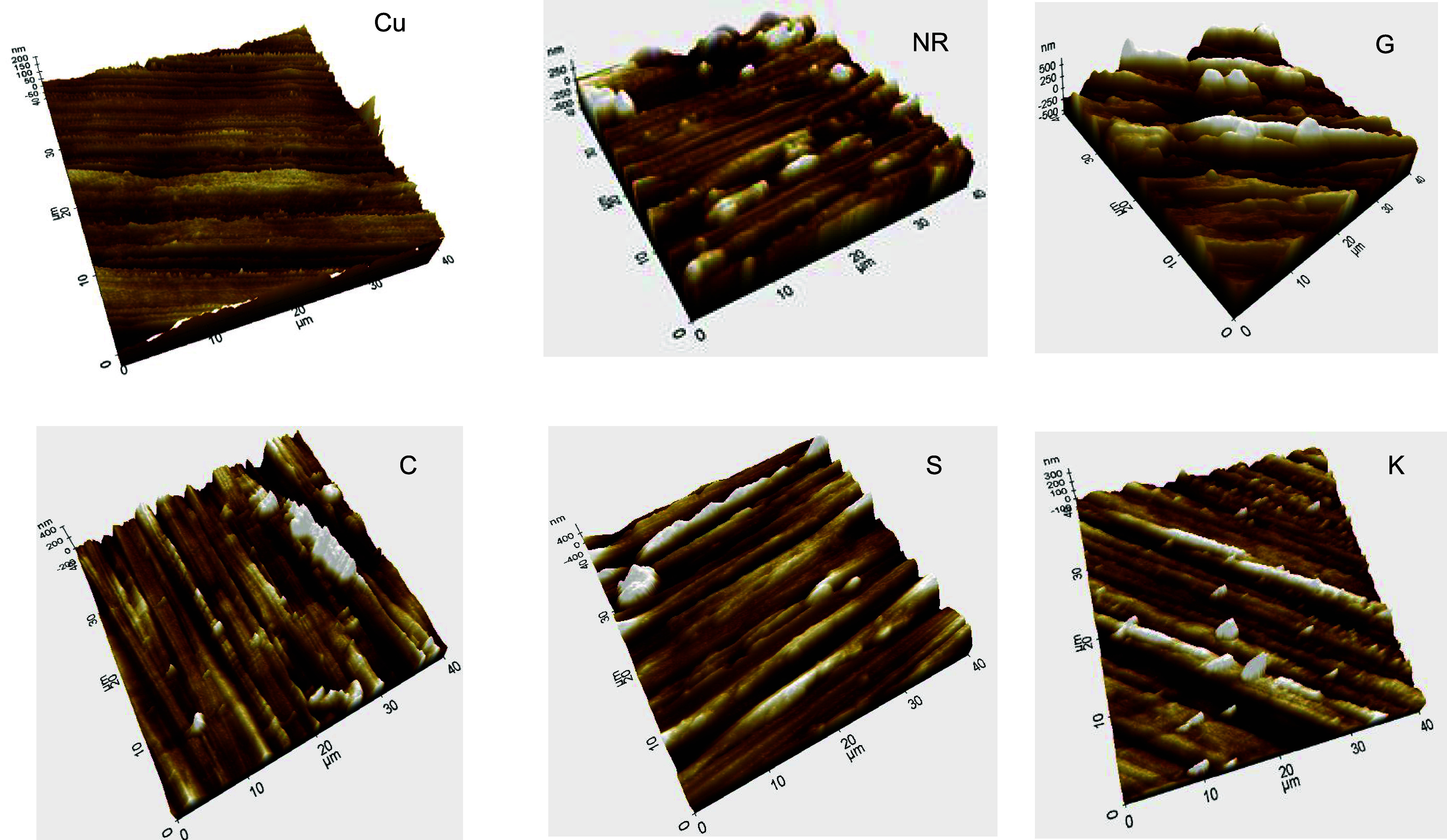
AFM images of the uncoated and B-pollen/SAM-modified copper electrodes, which were prepared using pollen harvested from different regions of Bingöl (B-pollen­(NR)/SAM (NR), B-pollen­(G)/SAM (G), B-pollen­(C)/SAM (C), B-pollen­(S)/SAM (S) and B-pollen­(K)/SAM (K)).

Figure 5 shows representative contact angle images and mean contact angles for the uncoated and coated electrodes. Compared to the uncoated electrode, the contact angle is higher for the coated electrodes. This increase can be attributed to the enhanced hydrophobicity of the coated surfaces. Upon examining Figure 5, SAM films prepared with pollen harvested from the northern regions and Karlıova exhibit higher contact angles. This suggests that the films are more compact and/or thicker, or that more hydrophobic moieties are oriented outward.

**Figure 5:**
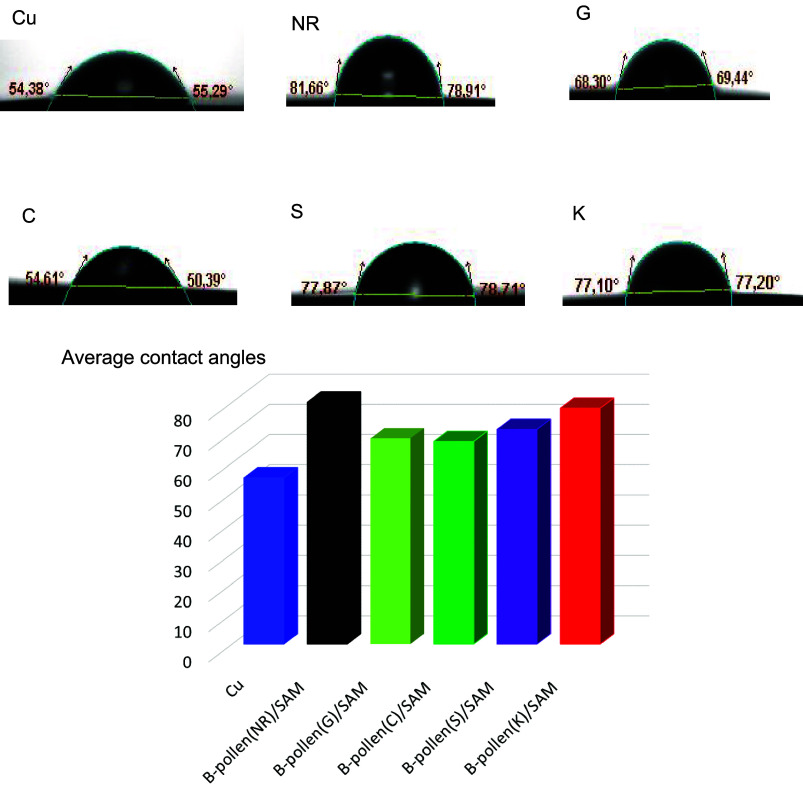
Contact angel images of the uncoated and B-pollen/SAM-modified copper electrodes, which were prepared using pollen harvested from different regions of Bingöl (B-pollen­(NR)/SAM) (NR), B-pollen­(G)/SAM (G), B-pollen­(C)/SAM (C), B-pollen­(S)/SAM (S) and B-pollen­(K)/SAM (K). Mean contact angles calculated from at least three measurements.

Research shows that the chemical makeup of bee pollen varies based on factors like plant source, geographic location, seasonal changes, and bee activity.^69,70^ The main components of pollen are proteins, carbohydrates, lipids, amino acids, polyphenols, and carotenoids.^70–72^ Pollen composition typically includes sugars (approximately 40%), proteins (around 23%), amino acids (roughly 10%), and lipids (approximately 10%). It mainly consists of two major groups: flavonoids and phenolic acids.^73^ Flavonoids are plant-derived polyphenolic structures with various subgroups such as flavones, flavonols, flavanones, flavanonols, flavanols, anthocyanins, chalcones, isoflavones, and neoflavonoids.^74^ The FT-IR analysis offered valuable insights into the chemical characterization of pollen samples collected from different regions of Bingöl. The results emphasized apparent regional differences in pollen composition and also revealed the presence of critical functional groups, highlighting the chemical complexity of these natural products. After partial purification with water, pollen extracts from five different regions (NR, G, C, S, K) were compared using their FT-IR spectra. It was clear that the spectral features of the B-pollen­(C) and B-pollen­(K) samples were very similar, while the extracts from B-pollen­(NR), B-pollen­(G), and B-pollen­(S) formed a separate group with more mutual resemblance (Figure 6). Across all samples, strong C–H stretching vibrations appeared at 2987–2974 cm^–1^ and 2904–2896 cm^–1^, indicating the presence of CH, CH_2_, and CH_3_ groups in phenolic acids and flavonoids. These bands confirm the abundance of these compounds across all regions. The bands between 1722 and 1651 cm^–1^ were linked to carbonyl (C=O) stretching, while those at 1407–1382 cm^–1^ related to C–H and O–H bending. Additionally, prominent bands at 1088–1057 cm^–1^ (C–O stretching) and around 885 cm^–1^ (C–C stretching) were observed in all regions (Figure 6).^75,76^ Notably, when regional variations were assessed, broad O–H stretching bands in the range of 3336–3328 cm^–1^ were particularly prominent in the spectra of B-pollen­(NR), B-pollen­(G), and B-pollen­(S). This feature indicates a higher concentration of polyphenolic compounds such as phenolic acids and flavonoids in these regions. In contrast, the B-pollen­(C) and B-pollen­(K) extracts showed distinct N–H stretching (3666–3658 cm^–1^) and C–N stretching (1236–1224 cm^–1^) bands, suggesting a higher presence of proteins and amino acids in these samples (Figure 6a).^77^ FT-IR analysis revealed that B-pollen samples exhibit significant chemical diversity influenced by their collection area, plant source, and environmental conditions. Additionally, FT-IR spectra of copper surfaces coated with partially purified pollen extracts were analyzed. These spectra confirmed the presence of organic compounds, including flavonoids, flavones, flavonols, flavanones, amino acids, and other compounds, within the pollen extracts. Strong N–H and/or O–H stretching bands (around 3500 cm^–1^), along with prominent C–H stretching (2931–2870 cm^–1^), and multiple bands between 1700 cm^–1^ and 875 cm^–1^, indicating carbonyl groups, bending, and stretching vibrations, demonstrated that the copper surfaces were effectively coated with the extract materials (Figure 6a). In summary, the FTIR spectra of pollen indicate that pollen composition varies according to the local flora. Although the pollen composition changes with regional flora, phenolic and flavonoid derivatives^78,79^ generally constitute a significant part of its makeup. Considering all this data, the structure of the bond can be schematized as Figure 6b.

**Figure 6:**
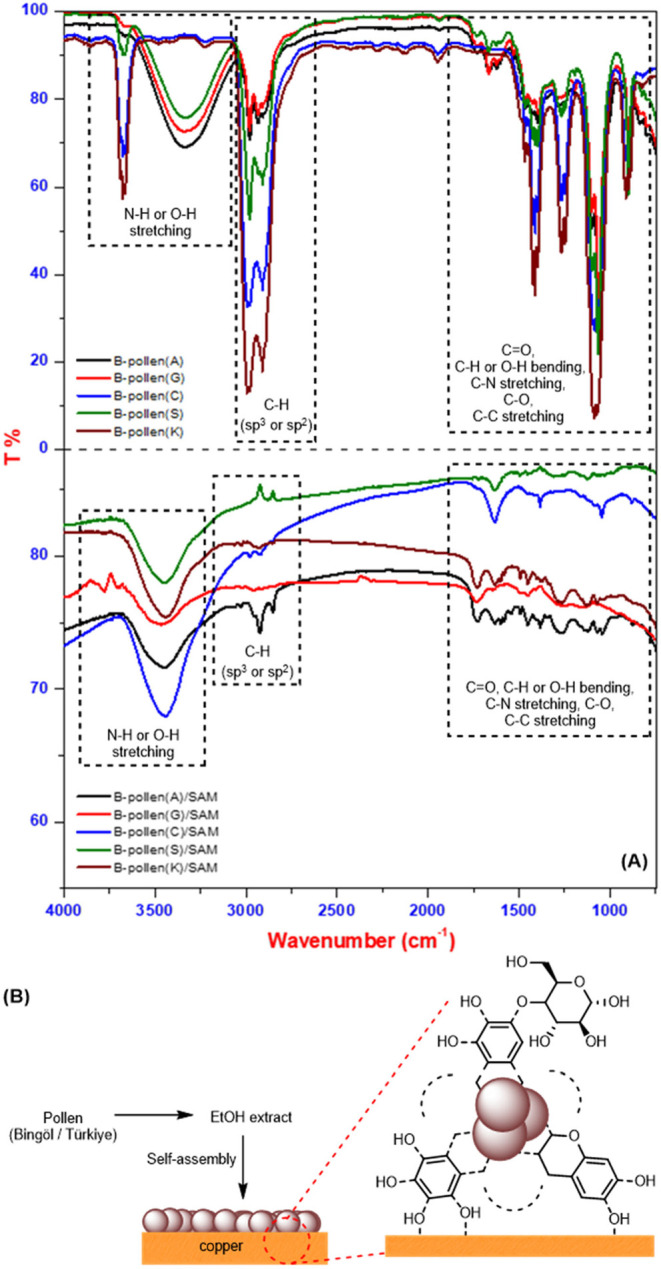
FTIR (KBr) spectra for B-pollen extracts and B-pollen-SAM films (A), proposed mechanism for pollen buildup on the Cu surface (B).

The surface analyses are consistent with the electrochemical studies and support the superior protective performance of the B-pollen­(K)/SAM film. In the next stage, the effect of exposure time on the corrosion performance of the pollen-SAMs was studied for this electrode.

### 3.2 The Effect of Exposure Time on the Corrosion Performance of the B-Pollen­(K)/SAM Coating

The variation of *E*_ocp_ with exposure time provides important information about the stability of the surface film, the initiation and progression of corrosion, and its effects on anodic and cathodic mechanisms.^80–83^ To examine this, the plot of *E*_ocp_–*t* for the uncoated and B-pollen­(K)/SAM-modified copper electrodes in 3.5% NaCl solution over 120 h is presented in Figure 7. The curves indicate that the *E*_ocp_ of the uncoated copper electrode shifts toward more positive potentials up to 24 h, then shifts back to more cathodic values and remains approximately −0.266 V (vs Ag/AgCl). Initially, a significantly nobler *E*_ocp_ value is observed for the B-pollen­(K)/SAM electrode, which shifts toward more cathodic potentials with increasing immersion time, and nearly remains stable after 24 h up to 120 h. This means that the film initially acts on anodic reaction, exhibiting a simple physical barrier effect.^62,84^ Subsequently, it predominantly affects the cathodic reaction by reducing the rate of the hydrogen evolution reaction.^85–87^

**Figure 7:**
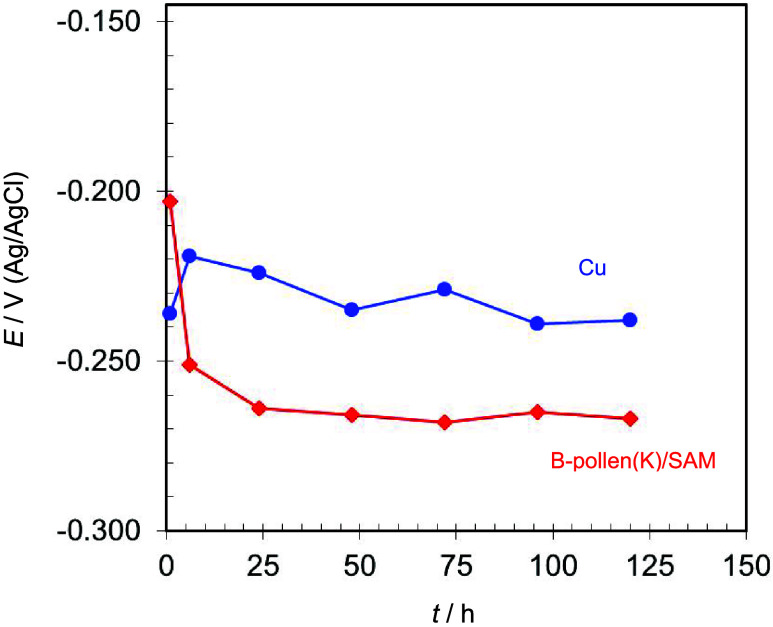
Plot of *E*_ocp_–*t* for the uncoated and B-pollen­(K)/SAM-modified copper electrodes in 3.5% NaCl solution over 120 h immersion.

The variation in the corrosion performance of the electrodes over time was monitored using the LPR technique, and the obtained *R*_p_ and *n*_R_% values are presented in Table 2. As clearly indicated by the data, the B-pollen­(K)/SAM film exhibits high corrosion resistance, with the *n*_R_% value remaining stable over time. Notably, even after 120 h, an inhibition efficiency of 98.7% was maintained, indicating high stability and protectiveness of the surface film. The corrosion resistance of uncoated copper initially increased relative to the 1 h (Table 1). This increase can be attributed to the accumulation of low-solubility corrosion products (such as chloride, hydroxychloride, or oxide compounds of copper) on the electrode surface, forming a physical barrier.^88–90^ Subsequently, a slight decrease in *R*_p_ can be explained by the increasing corrosion rate with exposure time.

**Table 2:** Electrochemical Parameters Derived from LPR Measurements for the Uncoated and B-pollen­(K)­SAM-Modified Copper Electrodes, Prepared Using Pollen Harvested from Karlıova Region of Bingöl, after Immersion in 3.5% NaCl for 1 h

	Uncoated Cu	Cu/B-pollen(K)/SAM	
*t* (h)	*R*_p_ (Ω cm^2^)	*R*_p_ (Ω cm^2^)	*n*_R_%
6	425	5357	92.1 ± 0.040
24	330	3704	91.1 ± 0.034
48	273	3333	91.8 ± 0.020
120	283	4202	93.3 ± 0.256

Nyquist plots of the uncoated and B-pollen­(K)­SAM-modified copper electrodes, prepared using pollen harvested from the Karlıova region, in a 3.5% NaCl solution after various exposure times are given in Figure 8. The electrodes exhibited two depressed semicircles; one in the high-frequency region and another in the low-frequency region. Additionally, in the low-frequency region, the observed straight line is attributed to diffusion, a phenomenon known as Warburg impedance. The high- to midfrequency time constant corresponds to charge transfer resistance and double-layer capacitance, whereas the low-frequency response is associated with the film resistance and the resistance of the accumulated corrosion products at the surface. The experimental EIS data related to these curves are given in Table 3. Apparently, the assembled pollen-SAM films significantly enhanced both charge transfer resistance and film resistance. The high protection efficiency maintained even after 120 h highlights the potential advantages of these films for practical applications.

**Table 3:** Electrochemical Parameters Derived from EIS Measurements for the Uncoated and B-pollen­(K)­SAM-Modified Copper Electrodes, Prepared Using Pollen Harvested from the Karlıova Region, Exposed to 3.5% NaCl for Various Times

	Uncoated Copper								B-pollen(K)SAM								
		CPE_dl_			CPE_f_					CPE_dl_			CPE_f_				
*t* (h)	*R*_ct_ (Ω cm^2^)	*Y* ^o^ (10^–6^/s*^n^* Ω^–1^ cm^–2^)	*n*_dl_	*R*_f_ (Ω cm^2^)	*Y* ^o^ (10^–6^/s*^n^* Ω^–1^ cm^–2^)	*n*_dl_	*W*	*R* (Ω cm^2^)	*R*_ct_ (Ω cm^2^)	*Y* ^o^ (10^–6^/s*^n^* Ω^–1^ cm^–2^)	*n*_dl_	*R*_f_ (Ω cm^2^)	*Y* ^o^ (10^–6^/s*^n^* Ω^–1^ cm^–2^)	*n*_dl_	*R*_p_ (Ω cm^2^)	*n*_R_%	χ2 × 10^–3^
6	6.7	346	0.884	338.2	5740	0.574	706.7	344.9	3538	270	0.661	3273	3464	0.658	6811	94.9 ± 0.036	493
24	14.2	470	0.856	379.0	3060	0.599	799.6	393.2	3005	280	0.710	2361	7130	0.905	5366	92.7 ± 0.028	563
48	15.4	460	0.590	395.6	2550	0.590	961.4	411.0	2743	190	0.788	3753	5000	0.865	6496	93.7 ± 0.021	545
120	6.1	370	0.910	289.2	2180	0.625	649.1	295.3	1699	485	0.741	5156	2360	0.746	6855	95.7 ± 0.021	10.5

**Figure 8:**
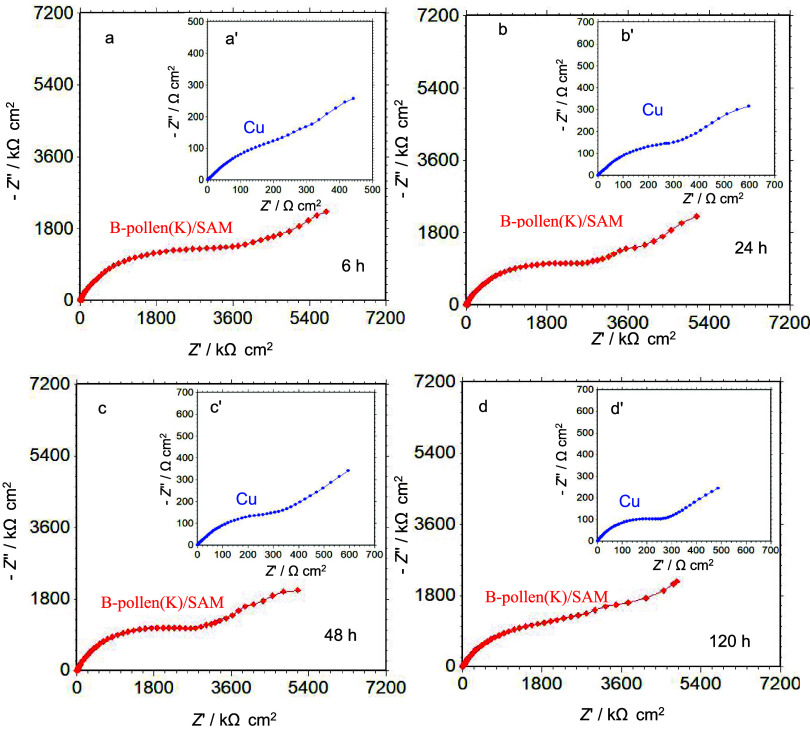
Nyquist plots of the uncoated and B-pollen­(K)­SAM-modified copper electrodes, prepared using pollen harvested from the Karlıova region, in %3.5 NaCl solution after various exposure times (a:6 h; b: 24 h; c: 48 h; d:120 h).

To enable a direct and accurate comparison among the data sets, the anodic PDP curves of the uncoated and B-pollen­(K)­SAM-modified copper electrodes, prepared using pollen harvested from the Karlıova region, were comparatively presented in a %3.5 NaCl solution after 1 and 120 h are given in Figure 9. This representation reveals that although some values appear close, measurable differences in electrochemical response are still present between the coatings prepared from pollens. The use of a unified scale, therefore, provides clearer visualization of these subtle but relevant variations. The relevant electrochemical data derived from these curves are shown in Table 4. The current densities at both 1 and 120 h decreased upon film formation.

**Figure 9:**
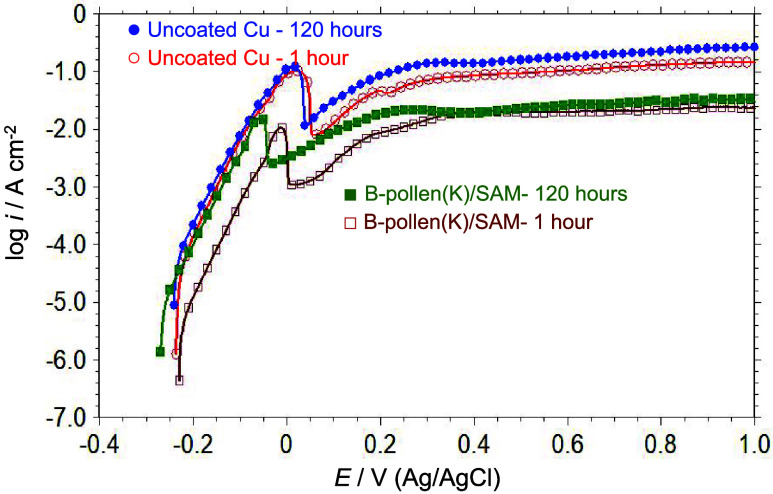
Anodic PDP curves of the uncoated and B-pollen­(K)­SAM-modified copper electrodes, prepared using pollen harvested from the Karlıova region, in %3.5 NaCl solution after 1 h (frame symbols) and 120 h (solid symbols) immersion.

**Table 4:** Electrochemical Parameters Derived from PDP Measurements for the Uncoated and B-Pollen­(K)­SAM Electrodes-Modified Copper, Prepared Using Pollen Harvested from Karlıova Region of Bingöl, Exposed to 3.5% NaCl for 1 and 120 h

Electrodes		*E*_corr_ (V, Ag/AgCl)	*i*_corr_ (μA cm^–2^)	*b*_a_ (mV dec^–1^)	*i*_0.100V_ μA cm^–2^	*i*_0.150V_ μA cm^–2^	η_i_%
Uncoated Copper	1 h	–0.236	70.75	60	6427.16	1002.97	
	120 h	–0.241	98.06	64	7934.94	1452.62	-
B-pollen(K)/SAM	1 h	–0.230	1.06 ± 0.267	59	544.98	82.79	98.5 ± 0.003
	120 h	–0.269	7.91 ± 0.500	60	3609.62	666.62	91.9 ± 0.025

The protective ability was over 90% even after 120 h, suggesting high protectiveness and stability of the B-pollen­(K)­SAM film on the copper surface. After 120 h of exposure, the *E*_ocp_ of the B-pollen­(K)­SAM-modified copper electrode was more negative than that of the uncoated electrode, indicating that the film mitigates corrosion with a predominance of the cathodic reaction. The anodic polarization curves in the Tafel region exhibited an almost parallel trend and the values of anodic Tafel slopes remained nearly constant after short (1 h) and long (120 h) immersion. The B-pollen­(K)­SAM film does not alter the reaction mechanism, and the effect is not dependent on immersion time.^61,91^ The film functions as a physical barrier that separates the metal surface from the corrosive environment. The anodic current densities, which are proportional to metal dissolution, decreased at the film-modified electrode, at fixed potentials (e.g., *i*_0.100V_ and *i*_0.150V_). These data also support the excellent protective performance of the B-pollen­(K)­SAM film.

The electrochemical test, which takes a maximum of 120 h, tests the efficiency of the self-assembled monolayer film, which can be obtained from natural sources such as pollen, in preventing corrosion, especially in situations where there is little degradation. Further studies are required to determine how efficient this film is in dynamic conditions, but the tests suggest its potential in conserving cultural heritage, microelectronic devices, and in environments with little corrosion. It is evident that the film is a significant improvement because it is both environmentally friendly and effective. As the composition of the film is dependent on its native sources, it is possible to alter its performance in the prevention of corrosion.

The CA curves of the uncoated copper and pollen-SAM-coated copper electrodes are presented in Figure 10. Initially, a rapid decrease in the current density was observed for the uncoated electrode. This decline is attributed to the formation of Cu^+^ and Cu^2+^ ions due to copper dissolution, which subsequently react with Cl^–^ ions to form low-solubility corrosion products such as CuCl(s) and CuCl_2_(s) that cover the electrode surface. However, since this corrosion product layer is unstable and does not provide effective protection, the current density remains high and fluctuates over time.^90–92^ In contrast, the B-pollen­(K)/SAM–modified electrode significantly reduced the current density. Moreover, the current remained nearly constant over time, indicating the stability of the protective layer. Thus, the pollen-SAM film provides excellent corrosion protection even after 120 h and remains highly stable on the electrode surface

**Figure 10:**
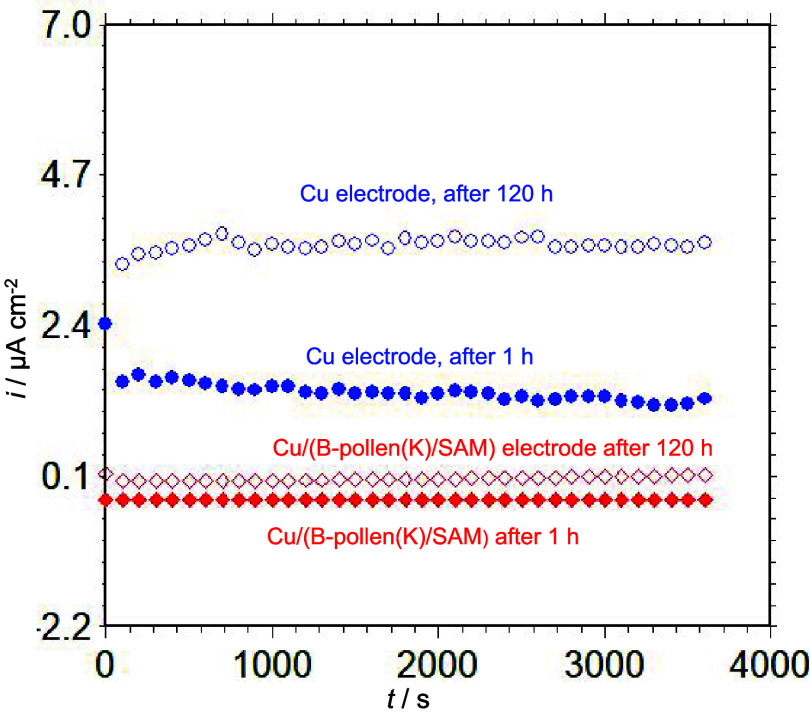
CA curves of the uncoated and B-pollen­(K)­SAM-modified copper electrodes, prepared using pollen harvested from the Karlıova region, in 3.5% NaCl solution, recorded after 1 and 120 h immersion.

The surfaces of uncoated and B-pollen­(K)/SAM-modified electrodes were analyzed using SEM, EDX and AFM analyses after exposure to 3.5% NaCl solution for 120 h. The data obtained are shown in Figure 11. As shown in Figure 11a, the bare copper surface reveals significant dissolution after 120 h, leading to the formation of large, deep pits on the surface. This observation indicates that the copper undergoes pitting corrosion, a well-known phenomenon in chloride-containing environments. In contrast, the B-pollen­(K)/SAM-modified copper electrode maintains a homogeneous and firmly adherent film even after prolonged exposure to the corrosive medium. This stable, protective layer significantly enhances the corrosion resistance of the metal. A similar observation was obtained from the AFM analysis. The EDX spectrum confirms the presence of elements such as C, N, and S, which originate exclusively from the pollen-derived molecules within the film. The EDX maps of the specific elements on the metal indicate a uniform, compact and/or thick film even after 120 h. The detection of chloride ions in the EDX analysis can be attributed to the formation of copper chloride compounds due to copper corrosion or the adsorption of chloride ions on the film surface.

**Figure 11:**
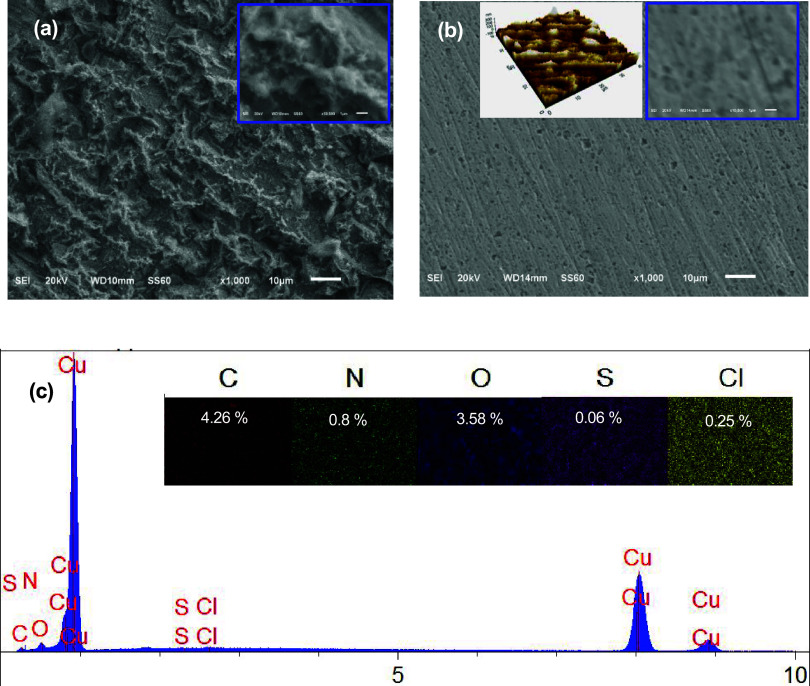
SEM images of the uncoated (a) and B-pollen­(K)/SAM-modified copper electrodes (b), AFM (on Figure 10b), EDX (c) spectrum and EDX mapping images of the B-pollen­(K)/SAM modified copper electrode after exposure to 3.5% NaCl solution for 120 h.

### 3.3 The Comparison of the Present Result with the Literature

A comparison of the protection ability of B-pollen­(K)­SAM electrodes-modified copper electrodes with the literature is presented in Table 5. The level of efficiency of the Karlıova pollen-based self-assembled monolayer (SAM) was assessed in relation to other significant SAMs. Even while other efficient biobased SAMs, such as Ginkgo biloba exocarp extract, show 97.8% efficiency,^6^ and phytic acid calcium reaches 92.5%,^7^ the optimally sourced Karlıova pollen-based SAM reaches a peak efficiency of 98.67%.^1^ In contrast to the conventional synthetic self-assembled monolayers (SAMs), such as triazines (TMTA) and triazoles (AMTa), which rely to a greater extent on the presence of one particular functional group, the efficiency of the monolayer film can be improved to the high 90% level (e.g., 91.0%^7^ for AMTa and 92.4% for TMTA^6^). Furthermore, high efficiencies up to 98.94%^2^ can be attained with long-chain alkanethiols, such as dodecanethiol, although this is done with the help of highly toxic and environmentally hazardous organic solvents.

**Table 5:** Comparison of the Protection Ability of B-Pollen­(K)­SAM Modified Copper Electrode with the Literature

SAM Film Name	Corrosive Environment	Inhibition Efficiency (%)	Refs
B-pollen(K)/SAM (Karlıova Pollen Extract)	3.5% NaCl	98.45	This Study
Ginkgo biloba exocarp extract SAM	3.0 wt % NaCl	97.86	^3^
Bingöl propolis SAM	3.5% NaCl	96.80	^4^
Phytic acid calcium (PAC) SAM	3.0 wt % NaCl	92.53	^5^
2,4,6-Trimercapto-1,3,5-triazine (TMTA) SAM	0.5 M NaCl	92.40	^6^
3-Amino-5-mercapto-1,2,4-triazole (AMTa) SAM	1.0% NaCl	91.00	^7^
Dodecanethiol SAM	Chloride-containing solution	98.94	^2^

More significantly, this monolayer, which is assembled in a unique synergistic manner, demonstrates an inhibition efficiency of over 90% after 120 h of continuous exposure to a highly aggressive 3.5% NaCl marine environment, a level of long-term stability rarely seen in simple synthetic monolayers without significant film degradation.

In addition to natural-product-based SAMs coatings reported in the literature, a wide range of SAM systems formed from synthetic thiols and other organic molecules^38,45,56,61^ have been extensively investigated for corrosion protection. Compared to these single-component SAMs, pollen-based SAMs present a distinct advantage due to their inherently complex chemical composition, containing various functional groups capable of interacting with the metal surface. This complexity may promote more effective surface coverage and contribute to a synergistic protective effect, where multiple molecular constituents collectively enhance barrier properties against corrosive species. These features highlight pollen-derived SAMs as a promising and unique alternative within the broader field of SAM-based corrosion protection.

It should be noted that pollen, as a complex chemical mix consisting of a variety of bioactive compounds (flavonoids, phenolics, proteins), inherently offers a variety of molecular dimensions, along with multiple electron-rich anchoring groups, which allow different molecules to coadsorb and methodically occupy surface defects to create a highly stable, tightly cross-linked physical barrier, which remains above 90% effective after 120 h in a harsh 3.5% NaCl solution.

## 4 Conclusions

B-pollen-SAM films were prepared on the copper surface using pollen harvested from different regions of Bingöl. The films were comprehensively characterized using advanced surface characterization techniques, such as SEM, EDX, AFM, and contact angle measurements. Their protective ability for copper corrosion in 3.5% NaCl solution was evaluated by *E*_ocp_–*t*, EIS, LPR and PDP measurements. The effect of exposure time on the protective ability of the film was investigated for the optimized film-modified copper. The films were also characterized by SEM, EDX and AFM measurements after the long exposure time. The following key findings are summarized:

- The SAM films of pollen harvested from different regions of Bingöl were successfully assembled on copper in water as a solvent. The appearance, physical and electrochemical properties of the films depended on the origin of the pollen.
- The SAM films significantly mitigate the corrosion rate of copper in 3.5% NaCl solution. The films inhibit corrosion through the predominance of the cathodic reaction.
- The protective ability of the films depends on the origin of the pollen, which is highest when Karlıova pollen is used.
- The B-pollen-SAM films were uniform, compact, and dense on the copper surface; the B-pollen­(K)/SAM film performed best.
- The B-pollen­(K)/SAM film retained its protective ability and stability, even after prolonged exposure (120 h).
- The films, particularly B-pollen­(K)/SAM, are well-suited for practical applications, considering that the stability of polymers, inhibitor films, and other organic coatings typically deteriorates over time.
- The successful implementation of sustainable biobased self-assembled monolayers in real-life industrial settings requires strict geographical tracking, as the source is the determining factor for the compactness and effectiveness of the resultant barrier.

## Supplementary Material



## Notes

Material includes Figure S1, which provides the representative fitting curves for the experimental EIS data (PDF)
