# Supplementary material for: Eco-Friendly Protection of Copper via Self-Assembled Monolayer Films: The Critical Effect of Pollen Source Region on Anti-Corrosion Behavior
Source: ACS Omega. 2026 Jun 19;11(25):38144–59. doi: 10.1021/acsomega.6c03873 (PMC13325392; doi:10.1021/acsomega.6c03873)
Supplement: Supplementary file 1 [file ao6c03873_si_001.pdf]

Suplemantary Data

Eco-friendly Protection of Copper via Self-Assembled Monolayer Films: The Critical Effect of Pollen Source Region on Anti-Corrosion Behavior

Ramazan Solmaz[*,a], Ece Altunbaş Şahin[*,b], Sinan Bayındır[c], Yeşim Aydın Dursun[d], İbrahim Halil Gecibesler[a, ı], , Mustafa Doğrubaş[e], Nevzat Çağlayan[f], İnan Dursun[f, ı], İbrahim Şahin[g], Handan Yüksel[c], İbrahim Y. Erdoğan[a], Gülfeza Kardaş[h]

[a]*Bingöl University, Health Sciences Faculty, Occupational Health and Safety Department, 12000, Bingöl, Türkiye*

[b]*Bingöl University, Genç Vocational School, Property Protection and Security Department, Civil Defense and Firefighting Program, 12000, Bingöl, Türkiye*

[c] *Bingöl University, Science and Letters Faculty, Chemistry Department, 12000, Bingöl, Türkiye*

[d]*Bingöl University, Graduate School of Natural and Applied Sciences, Chemistry Department, 12000, Bingöl, Türkiye*

[e]*Bingöl University, Graduate School of Natural and Applied Sciences, Occupational Health and Safety Department, 12000, Bingöl, Türkiye*

[f]*Bingöl University, Vocational School of Food, Agriculture, and Livestock, Plant and Animal Production Department, Beekeeping Program, 12000, Bingöl, Türkiye*

[g]*Bingöl University, Beekeeping Research, Development, Application and Research Center, 12000, Bingöl, Türkiye*

[h]*Çukurova University, Arts and Sciences Faculty, Chemistry Department, 01330, Adana, Türkiye*

[ı]*Bingöl University Central Laboratory Application and Research Center, 12000, Bingöl, Türkiye*

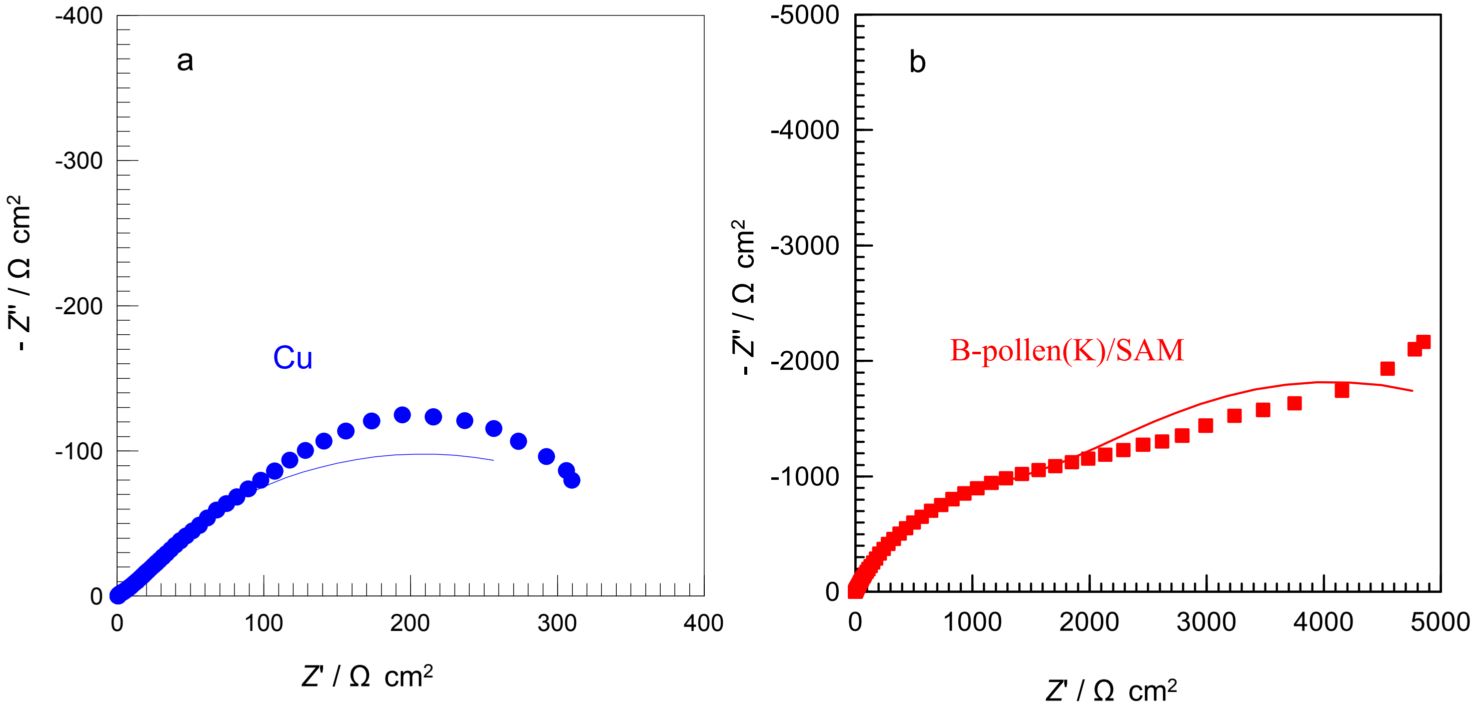

Figure S1. EIS fitting parameters for bare copper (a) and B-pollen(K)/SAM (b) after exposure to %3.5 NaCl solution for 1 h
